# Nurses' attitudes, beliefs and knowledge of sleep health in residential aged care: An integrative literature review

**DOI:** 10.1111/jan.16249

**Published:** 2024-05-31

**Authors:** Christopher J. Gordon, Tracee Fernandez, Emily Chen, Mariam Basheti, Matthew Rahimi, Bandana Saini

**Affiliations:** ^1^ Department of Health Sciences, Faculty of Medicine, Health and Human Sciences Macquarie University Sydney New South Wales Australia; ^2^ Centre for Sleep and Chronobiology Woolcock Institute of Medical Research Sydney New South Wales Australia; ^3^ Susan Wakil School of Nursing and Midwifery, Faculty of Medicine and Health University of Sydney Sydney New South Wales Australia; ^4^ Sydney Pharmacy School, Faculty of Medicine and Health University of Sydney Sydney New South Wales Australia

**Keywords:** attitudes, beliefs, knowledge, registered nurse, residential aged care, sleep

## Abstract

**Aim:**

To identify, synthesize and evaluate primary research on registered nurses' (RN) knowledge, attitudes and beliefs about sleep health and sleep health management of older adults living in residential aged care.

**Design:**

Integrative review.

**Data Source**s**:**

Medline, Embase and CINAHL databases from inception to September 2023.

**Review Methods:**

Databases were searched using a combination of key words, subject heading terms. All abstracts and full‐text articles were screened by two researchers. Qualitative synthesis of the included articles was conducted. Inductive content analysis was used to identify themes and analyse data.

**Results:**

A total of 923 abstracts were screened resulting in a final yield of 13 articles. Three themes were identified: (i) RN experience with sleep‐disturbed residents, (ii) the emotional burden of sleep disturbances on RN and, (iii) organizational barriers to promoting resident's healthy sleep. Inappropriate administration of benzodiazepines and psychotropic drugs to manage residents' sleep disturbances was a major issue and lack of resources in residential aged care to facilitate sleep. There were concerns on nursing activity that disturbed residents' sleep and striking a balance between facilitating sleep and meeting managerial expectations was challenging.

**Conclusion:**

This review identified that nurses' decision‐making has an integral role in the management of sleep health in residents in aged care. Whilst evidence‐based guidelines for managing sleep in residential aged care are available, there is a lack of translation to practice. Understanding RN perspectives is critical to improving sleep health models of care in residential aged care.

**Impact:**

This review found that RN are attuned to the implications of sleep disturbance in residential aged care but are constrained by current sleep health models of care.

**Patient or Public Contribution:**

Not applicable.

## 1 INTRODUCTION

Sleep is an essential biological function that is necessary for energy conservation, cell restoration, and consolidation of learning, memory, and general health (Crowley, 2011). Normal sleep patterns vary significantly across the lifespan, with more disrupted and less efficient sleep occurring in older individuals (>65 years), compared to younger individuals. In older people there are increased number of arousals during sleep, causing repeated awakenings, as well as a dramatic decrease in rapid eye movement sleep (REM), which normally accounts for 20%–25% of total sleep time. Decreased REM sleep has been shown to be associated with negative impacts on long‐term emotional wellbeing and memory retention (Feinsilver, 2003).

Sleep disturbance, a broad term that captures sleep complaints and sleep disorders, refers to any sleep disruption (including insufficient sleep or poor sleep quality/quantity) and is a major public health issue affecting the older population globally. The prevalence of sleep disorders has been estimated to be over 40% of the older population in the United States (Gordon et al., 2022) and 39% in Australia (Adams et al., 2017). Sleep disorders, which cause considerable sleep disturbance, are detrimental to quality of life, and increase the risk of developing physical and mental illnesses (Anderson & Bradley, 2013; Rod et al., 2011). There is clear evidence of a strong positive association between sleep disturbance and the development of chronic medical conditions, including type 2 diabetes, hypertension and stroke (Anothaisintawee et al., 2016; Grandner et al., 2016). In people with dementia, sleep disturbance may lead to worsening of behavioural and psychological symptoms of dementia (BPSD), such as restlessness and wandering (Wilfling et al., 2021). Sleep disturbances are also known to have a huge societal and economic impact with greater than $90 billion spent annually in the United States on health care costs alone (Huyett & Bhattacharyya, 2021). Considering that sleep health is a modifiable risk factor, improvements must be made to prevent and treat sleep disturbances and improve quality of life.

Globally, the prevalence of sleep disturbance in people with dementia in the community has been estimated to be 39% (Zhao et al., 2016), which contrasts with a prevalence rate of 70% in people with dementia in residential care homes (Webster, Costafreda Gonzalez et al., 2020). In comparison, the rate of insomnia symptoms and more broadly sleep disturbance has been reported to range between 15.9%–22.3% in the general population, with slightly higher rates in healthy older people (Gordon et al., 2022; Vitiello, 2006). Apart from medical and behavioural factors, environmental factors such as light exposure, noise, and the impact of nursing care can all negatively impact on resident's sleep in residential aged care (Kume et al., 2016). In older adults living in residential aged care, the main treatment option for health care professionals to manage sleep disturbance is medication (Harrison et al., 2020; Sluggett et al., 2022). Benzodiazepines, Z‐drugs and antipsychotic medications are the frequently used to treat sleep disturbance in older adults in residential aged care, however, these medications can cause increase drowsiness in older adults in residential aged care (Bourgeois et al., 2013). Unfortunately, long‐term use of these sedative‐hypnotic and antipsychotic medications to manage sleep is strongly associated with increased risk of falls (Kang et al., 2012), fractures (Wagner et al., 2004), and mortality (Patorno et al., 2017).

Despite these established risks, inappropriate and high rates of sleep medications (e.g. benzodiazepines) and psychotropic medications prescribing in RACF is consistently reported. Westbury and colleagues found evidence of increased frequency of PRN antipsychotic prescribing to manage behavioural and sleep problems associated with dementia, in 150 Australian RACF's which does not conform to the evidence‐based management requirements (Westbury et al., 2019). Given the adverse effects of psychotropic medications, non‐pharmacological strategies are recommended as first‐line interventions to manage sleep disturbances for older people living in residential aged care. Multicomponent intervention studies that examine the effectiveness of non‐pharmacological strategies such as physical activity, light therapy and mind–body practices have been shown to improve residents' sleep and decrease daytime somnolence and napping (Valenza et al., 2013; Wilfling et al., 2021). However, such non‐pharmacological strategies are underutilized in residential aged care due to organizational barriers such as a lack of standardized interventions and staff training (Westbury et al., 2019). A recent Australian parliamentary inquiry into the quality of care in residential aged care highlighted that there is a general lack of awareness and knowledge of sleep health among staff (Royal Commission into Aged Care Quality and Safety, 2019). High registered nurse (RN) workloads in aged care settings mean that RNs may not have sufficient time to manage sleep adequately, given the workforce constraints to provide care. There is now an increased emphasis on documentation and care coordination meaning RNs, who should play a vital role in the delivery and monitoring of residents' sleep health care in residential aged care are limited in their ability to undertake sleep health.

Whilst research has shown that nurses job satisfaction in aged care is high, staff experience levels and mix have found to be problematic, with nursing staff reporting poor skill mix to provide optimal care (Cameron et al., 2023; Peters et al., 2021). Staffing in aged care mainly comprises of nurses and personal care workers, and the quality of care is highly dependent on this workforce (Dawes & Topp, 2021). This could have an impact on the sleep health management of residents in aged care as non‐pharmacological approaches and strategies may require extra staffing resources as the workload is typically higher when compared to pharmacological management of sleep (Webster et al., 2022). Nursing staff have reported keeping residents awake during the daytime was challenging, and implementing sleep strategies overnight often take considerable time (Ludlow et al., 2021; Webster et al., 2022). In addition, organizational constraints have been identified as limiting the ability of registered nurses (RN) to provide care. This can lead to poor staff morale, lack of resources and training and ineffective care management in aged care (Hamiduzzaman et al., 2020; Kim & Park, 2017). This may have a negative impact on sleep health management in aged care due to limitations to autonomous care by RNs instituted by organizational controls.

RN, who are the most prevalent health care professional in aged care have the ability to identify sleep disturbances in residents as well as to address sleep concerns and promote good sleep health. Therefore, it is crucial that RNs are informed of evidence‐based practice and are provided with organizational support to facilitate the care needs of residents. To achieve this, evidence from successful interventions, the experiences and perspectives of key stakeholders and the current issues in sleep health management in residential aged care needs to be synthesized to design relevant strategies that could be used to improve sleep health in Australian residential aged care.

## 2 AIM

To date, a review of existing literature examining the perspectives and experiences of RN on sleep health and sleep health management specifically in setting of residential aged care is lacking. This integrative literature review aims to identify, synthesize, and evaluate existing research on RN's knowledge, attitudes and beliefs of sleep health and sleep health management in residential aged care.

## 3 METHODS

### 3.1 Study design

The integrative review methodology established by Whittemore and Knafl was deemed the most appropriate due to the inclusion of qualitative, quantitative and mixed‐method studies (Whittemore & Knafl, 2005). The review used the integrative review method to search, identify, and devise a final list of articles based on inclusion and exclusion criteria to provide a comprehensive review of research articles on the topic.

### 3.2 Search methods

A systematic search was conducted using three databases: Cumulative Index to Nursing and Allied Health Literature (CINAHL), Medline (Ovid) and Embase. CINAHL was chosen due to coverage of literature from nursing and allied health specialties. Medline was chosen due to the comprehensive health and medical research repository, and Embase provided a broadening of articles to complement CINAHL and Medline article yields. Searches of the databases was conducted from inception to September 2023, which would allow a more thorough search and exploration of the literature. Database specific search‐strategies including the use of MeSH headings and Boolean operators ‘AND’ and ‘OR’ were developed with assistance from a senior librarian. Numerous searches were conducted and refined to achieve a balance between the sensitivity and precision of the search. Search terms included a combination of subject heading terms, keywords and a comprehensive list of synonyms of the following: ‘nurs*’ ‘registered nurse’, ‘sleep*’, ‘aged care’, ‘residential aged care’, ‘nursing home’, and derivatives of ‘care’ as it relates to aged care ‘home care’, ‘long‐term care’ and ‘residential care’. Additional keywords including ‘attitudes’, ‘beliefs’ and ‘knowledge’ were included during the early stages of database searching but later removed due to an insufficient number of results across all three databases. The search was not limited by study design and publication date. To supplement the database search, reviews of reference lists of key studies, citation searches and hand‐searching for articles were conducted.

### 3.3 Inclusion/exclusion criteria

Residential aged care was defined as providing support and accommodation for older people who are unable to continue living independently in their own homes and who need ongoing help with everyday tasks. The inclusion criteria consisted of the following: (i) research participants were RNs working in residential aged care; (ii) research studies that focused on knowledge, attitudes and beliefs with respect to sleep health (including assessment, management, pharmacological/non‐pharmacological interventions, disorders, and disturbances); (iii) quantitative, qualitative, or mixed‐methods studies were included if written in English. The exclusion criteria consisted of the following: (i) participants who were RNs working in the hospital setting; (ii) participants who were not RNs (Assistants in Nursing, Enrolled Nurses carers, or nursing students) working in residential aged care settings [studies were included if the sample population included both non‐RN and RNs but data relevant to RNs only were extracted]; (iii) abstracts, commentary or reviews, and; (iv) non‐English publications.

### 3.4 Search outcomes

Titles and abstracts were screened by the authors (TF, MR) and articles which did not meet the inclusion criteria or met the exclusion criteria were removed. Any uncertainties during the screening and selection process were discussed with senior members of the research team (CG, BS). After the removal of duplicates and non‐English articles the remaining titles and abstracts were screened. All full text articles were read for suitability and excluded if not meeting the eligibility criteria.

### 3.5 Quality appraisal

The quality appraisal of included studies was conducted by the authors (TF, EC) and checked by senior members of the research team (CG, BS). To assess the clarity and comprehensiveness of the qualitative studies, the Critical Appraisal Skills Programme (CASP) checklist was used (Critical Appraisal Skills Programme (CASP), 2023). The CASP checklist comprises ten questions that aim to address three broad issues: validity of results, retrieval and analysis of results, and implications of the study. Three of the included studies were quantitative cross‐sectional research designs and the Joanna Briggs Checklist for Analytical Cross‐Sectional Studies was used to assess the strengths and limitations of the research methodology (Joanna Briggs Institute, 2017). This tool is an 8‐item checklist related to the study subjects and setting, validity and reliability of exposures and outcomes, confounding factors and statistical analysis. The mixed methods studies were critically appraised using the Evaluative Tool for Mixed Method Studies (Long, 2005). This tool consists of 7 review areas with key questions on the study evaluative overview, context, ethics, group comparability, data collection and analysis, and policy and practice implications.

### 3.6 Data abstraction

The purpose of data extraction and synthesize was to classify how the literature addressed the review aim and to identify and analyse key themes and concepts. The researchers immersed themselves in the content of the studies reviewed and used a coding process to outline emergent ideas based on the results section. The researchers discussed the key codes and potential themes, including senior authors for consensus‐building or to resolve disagreements for conflicting opinions until an agreed upon thematic derivation was achieved. The thematic analysis was done manually without software assistance (given the small number of studies identified). Data extraction also included authors, year, country, participants, objectives, study designs, data collection and findings. Two senior members of the research team (CG, BS) reviewed the data extraction table to ensure accuracy and validity (Braun & Clarke, 2006).

## 4 RESULTS

### 4.1 Study characteristics

**FIGURE 1:**
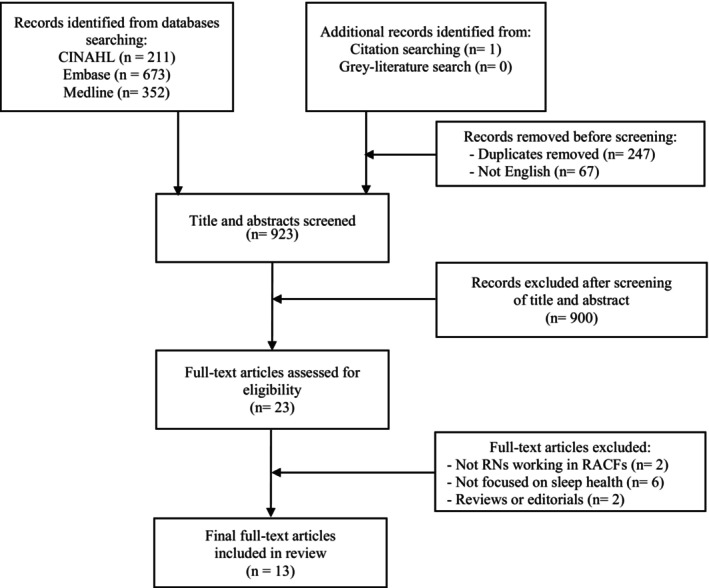
PRISMA flow diagram.

Overall, there were 923 titles and abstracts screened and 23 full‐text articles assessed for suitability. Eight studies were excluded for the following reasons: two did not have any registered nurse participants working in residential aged care; six studies did not focus on sleep health management and two studies were either abstracts, commentary or research reviews. Overall, 13 articles met the final inclusion criteria and were included in the integrative review (Figure 1).

The study designs included five qualitative methods (semi‐structured interview, focus group interview) (Anthierens et al., 2009; Ellmers et al., 2013; Ostaszkiewicz et al., 2016; Sawan et al., 2017; Webster, Powell et al., 2020), four were quantitative cross‐sectional studies (questionnaires, surveys) (Hughes et al., 2012; Janus et al., 2017; Wilfling et al., 2020; Wilfling et al., 2023), and four were mixed‐method studies (Eyers et al., 2012; Flick et al., 2010; Jacobson & Winograd, 1994; Nunez et al., 2018) (Table 1). The research studies were published between 1993 and 2023 and conducted in eight different countries: Australia (*n* = 2) (Ostaszkiewicz et al., 2016; Sawan et al., 2017), Belgium (*n* = 1) (Anthierens et al., 2009), Germany (*n* = 3) (Flick et al., 2010; Wilfling et al., 2020; Wilfling et al., 2023), North Ireland (n = 1) (Hughes et al., 2012), New Zealand (*n* = 1) (Hughes et al., 2012), The Netherlands (*n* = 1) (Janus et al., 2017), United Kingdom (n = 4) (Ellmers et al., 2013; Eyers et al., 2012; Nunez et al., 2018; Webster, Powell et al., 2020), and the United States of America (n = 1) (Jacobson & Winograd, 1994). Where data were provided, the mean age of RN participants was 39.7 years with an average of 12 years of RN experience. Additionally, study participants worked in nursing homes that were either government‐funded, privately owned, religious charities, or voluntary organizations.

**TABLE 1:** Characteristics of included studies.

Authors/Year	Country	Participants	Objectives	Study design	Data collection	Key findings
Anthierens et al. (2009)	Belgium	RN *n* = 33	To identify nurse's perception of using benzodiazepines in nursing homes and identify factors impacting on nurses’ role with benzodiazepine	Qualitative interviews	Three focus group interviews (5–8 RNs per group) Ten semi‐structured interviews with RN	Three factors had an impact on nurse's perceptions in benzodiazepine usage: individual attitude and perceptions, knowledge and organisational factors
Ellmers et al. (2013)	United Kingdom	Nursing staff *n* = 39^a^ Residents *n* = 38	To explore sleep management in 24 h care home environment	Qualitative interviews and ethnographic observations	Semi‐structured interviews 48 h of observations conducted over 2–3 weeks	Night staff had contradictory demands balancing the individual choices of resident's sleep and the care home management requirements
Eyers et al. (2012)	United Kingdom	Nursing staff *n* = 50^a^ Managers *n* = 10 Residents *n* = 145	To identify the determinants of poor sleep in care homes	Mixed methods interviews field observations	Semi‐structured interviews 250 h of observational data and field notes evaluating residents 2‐week diaries	Regular surveillance by RN's and care assistants at night seriously impedes sleep quality of older people in care homes
Flick et al. (2010)	Germany	Nurse *n* = 17 NA *n* = 5 Caregivers *n* = 10	To determine if nurses are aware that daytime activities can improve sleep quality at night in nursing homes	Mixed methods interviews survey	Interviews with staff from 10 nursing homes Survey of activity levels in nursing homes	Nurses identified professional standards as a barrier to managing sleep disturbances. Nurses’ knowledge was poor about sleep health and was developed on local experiences rather than evidence‐based practice
Hughes et al. (2012)	Northern Ireland New Zealand	RN *n* = 186 NM *n* = 61	To measure treatment culture (beliefs, values, and normative practices associated with medication prescribing and administration) in two samples of nursing homes	Quantitative cross‐sectional	Questionnaire	Organisational culture influences the patterns of use of psychotropic medications in nursing homes. Differing views about the use of antipsychotic medications by NM's and RN's may pose issues on the writing and enacting of policies
Jacobson & Winograd (1994)	United States of America	Nursing staff *n* = 14^a^ Physicians *n* = 3 Residents *n* = 14	To explore the difference among patients, nurses and physician's perceptions of psychoactive medications and management of night‐time disorders	Mixed methods interviews mini‐mental status examination	Structured interview with nurses, patients and physicians Mini‐Mental Status Exam for patients	Disagreements between patients, nursing staff and physicians suggests that nighttime disorders and their management are perceived differently. Poor communication may explain these differences
Janus et al. (2017)	The Netherlands	RN *n* = 31 Nurse asst *n* = 50	To explore the determinants of nurses’ and nursing assistants request for antipsychotics for people with dementia	Quantitative cross‐sectional	Online questionnaire	Nurses and nursing assistants with a lower job satisfaction were more likely to call for antipsychotic medications. Having more positive beliefs about treatment effects and feeling of being in control toward asking for antipsychotics were positively associated with intention to call
Nunez et al. (2018)	United Kingdom	Nurse *n* = 19 Family carers *n* = 16	To explore the current practices and challenges in night‐time care for people with dementia living in care homes in the United Kingdom	Mixed methods interviews survey	Two focus group discussions (FGD) conducted with family carers (*n* = 5) and care staff (*n* = 12). Online surveys supplemented the family carer FGD data. Informal interviews during a night shift to facilitate night staff involvement	Eight key themes in the management of sleep disturbance in people with dementia living in care homes: current night‐time care practices, dissonance between perceived causes of sleep disturbances, inconsistencies in treatment options, insufficient staffing levels, working relationships between shifts, nurses' burden and responsibility, communication as a critical challenge, connecting with residents and one overarching theme of balance
Ostaszkiewicz et al. (2016)	Australia	Nurse *n* = 6 (comprised of DON *n* = 4; CNE *n* = 2) EN *n* = 6 NA *n* = 6	To describe nurses’ and personal care workers’ beliefs and experiences of providing continence care at night in residential aged care facilities	Qualitative‐ interviews and field observations	In‐depth interviews conducted by one researcher Non‐participant field observations	Staff decisions about night‐time incontinence care were influenced by personal and experiential knowledge about residents, work pressure to reduce pressure injuries and policies/regulations
Sawan et al. (2017)	Australia	RN *n* = 8 CNC *n* = 1 NP *n* = 1 EN *n* = 2 Manager *n* = 8 NA *n* = 5 GP *n* = 8 Pharmacist *n* = 6 SD *n* = 1	To explore the key dimensions of organizational climate and their subsequent influence on the use of psychotropic medicines	Qualitative‐ interviews	Forty semi‐structured interviews. The median duration of the interviews was 50 min	Factors such as staffing, managerial expectations and teamwork among staff members influenced the appropriate use of psychotropic medicines
Webster, Powell et al. (2020)	United Kingdom	RN *n* = 4 Nurse asst *n* = 14	To explore staff experiences of sleep disturbance in residents with dementia	Qualitative‐ interviews	One‐to‐one semi‐structured interviews	Staff reported sleep disturbances to negatively impact not only the residents themselves but also other residents, staff, and occasionally their relatives. Some of the strategies used by staff to cope with sleep disturbances might be counterproductive
Wilfling et al. (2020)	Germany	RN *n* = 77 Nurse asst *n* = 34	To determine nurses’ burden associated with sleep disturbances of nursing home residents with cognitive disorder	Quantitative cross‐sectional	Three standardised questionnaires The Sleep Disorder Inventory Scale was used to assess nurses’ burden	Nurses are regularly confronted with residents’ sleep disturbances during night shifts, causing burden. The most common source of knowledge about interventions to avoid sleep disturbance was personal working experience, followed by continuous nursing education and nursing training
Wilfling et al. (2023)	Germany	RN *n* = 192 Nurse asst *n* = 47	To understand the attitudes and knowledge of nurses working at night towards sleep of nursing home residents	Quantitative cross‐sectional	Survey of attitudes and knowledge about sleep and pharmacological interventions for sleep	Nurses working night shift in RACFs rated sleep disturbance as important and that assessment was nurses’ responsibility. Most nurses reported that they have not received sleep management training

Abbreviations: CNC, Clinical Nurse Consultant; CNE, Clinical Nurse Educator; DON, Director of Nursing; EN, Enrolled Nurse; GP, General Practitioner; NM, Nurse Manager; NP, Nurse Practitioner; Nurse asst, Nurse Assistant; RN, Registered Nurse; SD, Specialist Doctor.

^a^ Study did not obtain specific degree affiliation, thus, unable to distinguish exact role of registered nurse participants.

Twelve out of thirteen studies included participants in addition to RNs, including physicians, nurse assistants, residents, caregivers, and allied health professionals. Two studies did not explicitly outline the role and/or qualifications of nursing staff participants (Ellmers et al., 2013; Jacobson & Winograd, 1994). Four studies investigated the use of pharmacological interventions including antipsychotics and benzodiazepines (Anthierens et al., 2009; Hughes et al., 2012; Janus et al., 2017; Sawan et al., 2017), while two studies explored the influence of treatment culture and organizational factors on nursing sleep health management practices (Hughes et al., 2012; Sawan et al., 2017). Studies also focused on the management of sleep disturbances (Ellmers et al., 2013; Webster, Powell, et al., 2020; Wilfling et al., 2020; Wilfling et al., 2023), determinants of poor sleep (Eyers et al., 2012) and current nursing sleep practices (Nunez et al., 2018; Ostaszkiewicz et al., 2016).

The quality of the study research design was assessed through quality appraisal tools for the different study designs. Overall, it was found that there may have been bias in the qualitative studies that was not addressed (Table 2). Moreover, the quantitative and mixed‐methods studies were generally conducted in a methodologically rigorous manner, but there remained some limitations that were not sufficiently addressed (see Tables 3 and 4).

**TABLE 2:** Critical appraisal of qualitative studies.

Authors, year	Aim/s stated	Appropriate methodology	Appropriate study design	Appropriate recruitment strategy	Appropriate data collection	Potential bias addressed	Ethical issues considered	Appropriate data analysis	Appropriate discussion of findings	Implications addressed
Anthierens et al., 2009										
Ellmers et al., 2013										
Ostaszkiewicz et al., 2016										
Sawan et al., 2017										
Webster, Powell et al., 2020										

*Note*: Green–yes; Yellow–not adequately addressed/not applicable; Red–no.

**TABLE 3:** Critical appraisal of quantitative studies.

Authors, year	Inclusion criteria	Subject and setting described	Exposure measured	Criteria for measurement of condition	Confounding factors identified	Strategies to deal with confounding factors	Outcomes measured in valid and reliable way	Appropriate statistical analysis
Hughes et al., 2012								
Janus et al., 2017								
Wilfling et al., 2020								
Wilfling et al., 2023								

*Note*: Green–yes; Yellow–not adequately addressed/not applicable; Red–no.

**TABLE 4:** Critical appraisal of mixed‐methods studies.

Authors, year	Bibliography details stated	Purpose stated	Key findings	Evaluative summary stated	Study design	Setting	Sample	Outcomes	Ethics	Comparable groups	Data collection	Data analysis	Potential bias	Implications addressed
Jacobson & Winograd, 1994														
Flicket al., 2010														
Eyers et al., 2012														
Nunez et al., 2018														

*Note*: Green–yes, Yellow–not adequately addressed/not applicable, Red–no.

Across the 13 studies included in this review, thematic content emerged naturally under three broad concepts: (i) RN' practice and perceptions of sleep‐disturbed residents, (ii) the emotional burden of sleep disturbances on RN, and (iii) organizational factors and sleep health.

### 4.2 RN practice and perceptions of sleep‐disturbed residents

#### 4.2.1 RN' perceptions of residents' sleep disturbances

In the study by Webster et al., nursing staff's opinions on how sleep disturbances manifest in and impact residents with dementia, other residents, and staff were investigated (Webster, Powell et al., 2020). The findings show that disturbed night‐time sleep had a negative effect on residents' behaviour or mood the next day. Not only were residents more irritated and aggressive toward staff, but disturbed sleep affected their ability to eat and drink, participate in physical activities, and take their regular medications with one participant stating, *‘…we're trying to make sure he has taken the medication covertly, (that) he has the food’*. Other participants commented on the physical effects on residents experiencing sleep disturbance: *‘she doesn't sleep in her bed and her legs swell sometimes’ ‘especially with that kind of pacing day and night, since he is not sleeping well. So he's like using much energy and that contributed to weight loss’*.

#### 4.2.2 Common misconceptions about residents' sleep

There were common misconceptions about the usage of antipsychotics and benzodiazepines. Studies reported that RNs knowledge about benzodiazepines and sleep was poor, dated and factually incorrect (Anthierens et al., 2009; Hughes et al., 2012; Jacobson & Winograd, 1994; Janus et al., 2017; Sawan et al., 2017; Wilfling et al., 2020; Wilfling et al., 2023). This was typified with one participant stating: ‘*Sleeping pills do not give that many side effects’* and another, ‘…*fall incidents are more associated with the mobility of the older in general, it is not directly linked with sleeping pills*’ (Anthierens et al., 2009).

Interview data revealed a gap between the existing awareness of sleep disorders and the consequences on a practical level (Flick et al., 2010). Evidence suggested that RNs did not believe in influencing residents' sleep–wake behaviour through activities and structured routines. Instead, daytime activities were considered entertainment without a therapeutic use for sleep improvement. On a similar note, some strategies used by nurses to address sleep disturbances were counterproductive. Webster and colleagues reported that residents were often provided caffeinated tea at night, which may aggravate sleep disturbances (Webster, Powell et al., 2020).

#### 4.2.3 Implementing pharmacological interventions to manage sleep disturbances

Reports of inappropriate medication administration were also identified (Janus et al., 2017). Nurses and nursing assistants requested antipsychotics for residents with dementia regularly with 57% of nurses believing that administering antipsychotics resulted in less psychological burden for staff and 44% reporting that antipsychotics lowered nursing workloads. In addition, 54% of nurses agreed that antipsychotics resulted in physiological calmness of the resident; however, a majority either disagreed or were neutral about the positive effect of antipsychotics on residents' quality of life. Several studies also suggested that sleeping pills are the obvious method that nurses to utilize to address sleep problems (Anthierens et al., 2009; Jacobson & Winograd, 1994; Nunez et al., 2018). On a similar note, it was highlighted that benzodiazepines were used in a such a routine way that there was little attempt to undertake a re‐assessment or consideration of potential alternatives: ‘*We do not think about sleep medication. People have been taking their sleeping tablets for years*. *There is no evaluation of whether it is still necessary or not*.’ (Anthierens et al., 2009).

### 4.3 The emotional burden of sleep disturbances on RN

#### 4.3.1 Stress and guilt experienced by RN

The participants in Webster et al. described how dealing with those who had sleep disturbances made them feel stressed and guilty due to their conflicting desire and ability to provide care to other residents (Webster et al., 2020). A similar concept was found by Wilfling et al. that despite nurses claiming to know about interventions to manage sleep disturbances, the emotional burden caused by sleep disturbances was common with 78.4% stating to be regularly confronted with residents' sleep disturbances at night and 80.1% stated being mildly‐severely emotionally distressed (Wilfling et al., 2020).

#### 4.3.2 Obstacles in the professional standards

One of the challenges to facilitating good sleep is night‐time care. Several studies highlighted that staff had contradictory demands between the individual choices of residents about their sleep and the expectation of staff to regularly check on residents sleeping at night (Ellmers et al., 2013; Eyers et al., 2012; Ostaszkiewicz et al., 2016; Webster, Powell et al., 2020). Research revealed that the effect of regulatory frameworks on residents' night‐time care caused staff to feel pressured to minimize the occurrence of adverse events such as pressure injuries and falls, therefore wakening residents overnight and disturbing sleep (Eyers et al., 2012).

### 4.4 Organizational barriers and sleep health

Overall, it was reported that nurses recognized sleep hygiene was an important component of improve good sleep health but acknowledged that they lacked expertise in applying it and considered organizational factors to be major barriers restricted the application into sleep health practices. This was mainly related to lack of resources, communication problems and embedded treatment cultures, preclude the implementation of sleep hygiene.

#### 4.4.1 Lack of resources

Several reports highlighted a lack of resources to better manage residents sleep disturbance in residential aged care (Anthierens et al., 2009; Ellmers et al., 2013; Eyers et al., 2012; Flick et al., 2010; Jacobson & Winograd, 1994; Webster, Powell et al., 2020; Wilfling et al., 2020; Wilfling et al., 2023). In particular, RNs were constrained by the organizational aspects, rather than knowledge about sleep, and especially using evidence‐based practice to facilitate and promote sleep. Sawan et al. used semi‐structured interviews to explore the link between organizational climate and the use of psychotropic medications in nursing homes and found that RNs thought psychotropic medications were a *‘necessary evil’* to compensate for lower staffing levels at night and accordingly RNs used medication as a *‘coping mechanism*’ to manage residents with insomnia and behavioural/psychological symptoms of dementia (Sawan et al., 2017). This aligns with findings that show benzodiazepines are associated with the smooth and efficient running of residential aged care (Anthierens et al., 2009). There was also consensus that staffing levels directly affected residents' sleep–wake patterns with increased staff levels improving resident's daytime engagement and lowering the requirement for psychotropic medications. Moreover, staffing did not always facilitate giving residents a choice over sleep times (Ellmers et al., 2013). Lower levels of nursing staff directly affected the time that residents went to bed and resulted in some residents having to go to bed earlier desired.

#### 4.4.2 Communication and collaboration

Two studies reported that when RNs are concerned about the sleep health of residents, they convince doctors to take appropriate action by either prescribing sleeping medication or evaluating current medication regimens (Anthierens et al., 2009; Janus et al., 2017). Nurse‐doctor relationships can also pose a barrier to nurses raising concerns due to the unequal balance of power between the two professions. An older study found that poor communication between nurses and physicians can lead to an inaccurate perception of residents' sleep problems which may lead to inappropriate medication management (Jacobson & Winograd, 1994).

#### 4.4.3 Treatment culture

In a study conducted across Northern Ireland and New Zealand, found that RNs in both countries were more likely than nurse managers to incorrectly report that antipsychotic medication (haloperidol) should be used for short‐term insomnia, indicating a treatment culture related to the beliefs and practices associated with prescribing medications for sleep (Hughes et al., 2012). However, New Zealand and Northern Island nurses disagreed about the order of implementing medication or social activities to improve sleep with New Zealand RN's wanting to use social activities prior to *as needed* (PRN) psychotropic medications.

## 5 DISCUSSION

Older adults living in residential aged care are more likely to experience sleep disturbances due to combination of poor physical and psychological health, significant co‐morbidities, polypharmacy, environmental factors and institutionalization. This integrative review sought to synthesize research studies that have explored the knowledge, attitudes and beliefs of RNs on sleep health and sleep health management in residential aged care. The research studies explored treatment modalities, dementia‐specific sleep disturbances and organizational culture, which provided a broad lens view of nursing considerations for residents living in residential aged care. Three main themes emerged from the studies related to RNs practice and perceptions of resident's sleep, the emotional burden experienced by the RNs and the impact of organizational factors on resident's sleep health. It was also found that there is a lack of data about how RNs evaluate and initiate pharmacological and non‐pharmacological sleep health interventions in residential aged care and the challenges caring for residents with sleep disturbances.

The first theme to emerge from studies reviewed in this review was nurses' practice and perceptions of sleep‐disturbed residents. Changes in residents' behaviour, mood and physical health were observed however, there was obscurity around the factors influencing nursing practice, in particular the assessment, communication and management of sleep disturbance in residential aged care. Previous research has suggested that sleep education is poorly integrated into undergraduate nursing curricula, which often results in RNs using their own experiences with sleep and/or their clinical experience to address patients sleep health problems (Huang et al., 2018; Ye et al., 2013). There was associative evidence from this review of the nursing care to address sleep disturbances which was often counterproductive and driven by a lack of evidence‐based knowledge (Anthierens et al., 2009; Ellmers et al., 2013; Jacobson & Winograd, 1994; Webster, Costafreda Gonzalez et al., 2020; Wilfling et al., 2023). If nursing practice for sleep health were to improve their need to be changes in pre‐registration education in addition to continuing professional development for RNs on sleep health and sleep health management. Considering that nurses may care for residents with dementia, it may also be beneficial to receive dementia‐specific education to manage behavioural and psychological symptoms of dementia. Moreover, the common practice of administering psychotropic medications for sleep disturbances should be evaluated closely as the studies showed that staff workload and stress was positively associated with the decision to request for PRN medications (Ellmers et al., 2013; Webster, Powell et al., 2020). This raises questions as to the appropriateness of psychotropic medication prescribing and whether it benefits the resident or nursing staff. Empowered staff and manageable workloads may be the key to more appropriate use of psychotropic medications and better sleep health for residents.

It was found that the emotional burden of sleep disturbances on RNs was high and that RNs experienced guilt and are often feel overwhelmed when caring for RACF residents. RN were aware that they should address residents' sleep problems more systematically and employ non‐pharmacological interventions first to align with the evidence. While doing so would provide RNs with increased job satisfaction, many felt that they were not working in an *‘ideal setting’* and they were often overwhelmed by work pressure, which promotes medicalisation and pharmaceuticalisation (Coveney et al., 2019). Similarly, RNs highlighted challenges associated with regular night‐time care with many emphasizing the added pressure by facility managers to prevent complications (Eyers et al., 2012; Nunez et al., 2018; Ostaszkiewicz et al., 2016). In order to effectively meet the sleep health needs of residents, systemic problems in aged care must be acknowledged and addressed. Adequate resources in residential aged care are arguably one of the principal causes of substandard care, and despite several reforms, there still remains a lack of funding, poor staffing ratios, and poor skill mix and training (Royal Commission into Aged Care Quality and Safety, 2021).

While RNs had positive intentions to promote good sleep health in residents, there were organizational barriers that influenced their decision‐making and nursing practice. For instance, when there were less staff then the evitable rush to complete tasks becomes normalized resulting in less resident daytime stimulation and day routines dictated by nursing routines rather than evidence‐based sleep practices (Flick et al., 2010). Activity, time, and social interaction with residents, particularly those living with dementia are critical in promoting strong sleep–wake behaviours and further research into time factors and nursing care provision is required to improve residents' sleep. Other organizational factors such as staff and resource constraints, organizational support, workload, consistency and unity of policies and guidelines, and availability of educational programs all have implications on nurses' capacity to promote good sleep (Alaseeri et al., 2021). In addition, inter‐disciplinary communication and collaboration between nurses and doctors have traditionally been hierarchical in nature with most of the decision‐making vested in the doctors. This was evident when benzodiazepines were prescribed by a doctor despite the RNs observation and interpretation of the residents sleep, RNs feel powerless to discuss the *‘inappropriate’* prescription (Anthierens et al., 2009). In order to improve the quality of resident sleep care, there needs to be an impetus of change the long‐established professional norms and boundaries between health professions. A teamwork approach should be recognized taking into consideration that the relationship between doctors and nurses is complementary and nurses are partners in resident care. Similarly, the idea of treatment culture, which focuses on the beliefs, values, and normative practices associated with medication prescribing and administration is common in healthcare settings are often inappropriate for residents living in aged care. Despite an extensive evidence base, psychotropic medications are still used in residential aged care to manage sleep disturbance (Harrison et al., 2018).

There were some limitations in this integrative review. The review was limited to studies in English and there may have been studies published in other languages that were relevant. The generalisability of the findings may also be constrained as studies from different countries with distinct nursing regulations in residential aged care may have impacted on the way care was delivered. The data analysis used inductive content analysis and the synthesis was based on understandings of other researchers' interpretations. This review only focused on RNs and other types of nurses or health professionals may have different experiences which could impact on resident's sleep health.

## 6 CONCLUSION

Sleep disturbances in residents living in residential aged care are a major problem, necessitating pragmatic evidence‐based solutions. It is clear from this integrative review there remains an over‐reliance on medications as first‐line management of resident's sleep disturbances. There is strong evidence‐based non‐pharmacological approaches which improve the health and well‐being of residents which are not currently being implemented. Unfortunately, organizational constraints related to a lack of resources, inadequate staffing and poor nurses' knowledge, heavy workload and staffing ratios contributes significantly to the continued pharmacological approach. Moreover, resident's sleep health in aged care remains suboptimal due to a complex interplay of factors pertaining to nursing practice and organizational influences. If optimal, person‐centred care that seeks to not only improve sleep health but to address other needs such as improvements in quality of life, RNs have the potential to have a significant impact on the lives of residents experiencing sleep disturbances. RN are well‐positioned to identify and address sleep health problems in residents living in a variety of aged care settings. There should be a proactive approach to improving the sleep health of residents. Future studies should explore how nurses assess and conduct sleep assessments, implement and evaluate sleep health interventions in residential aged care.

## FUNDING INFORMATION

Funding for this review was provided by the National Health and Medical Research Council: National Centre for Sleep Health Services Research—Positioning Primary Care at the Centre of Sleep Health Management Centre of Research Excellence, GNT1134954.

## CONFLICT OF INTEREST STATEMENT

All authors have no conflict of interest related to this review.

### PEER REVIEW

The peer review history for this article is available at https://www.webofscience.com/api/gateway/wos/peer‐review/10.1111/jan.16249.

## Data Availability

All data are available from the authors upon request.
